# Effects of Peroxisome Proliferator-Activated Receptor-Gamma Agonists on Cognitive Function: A Systematic Review and Meta-Analysis

**DOI:** 10.3390/biomedicines11020246

**Published:** 2023-01-18

**Authors:** Hongfei Zhong, Rulin Geng, Yu Zhang, Jingwen Ding, Miao Liu, Shengfeng Deng, Qiuyun Tu

**Affiliations:** Department of Geriatrics, The Fifth Affiliated Hospital of Sun Yat-Sen University, Sun Yat-Sen University, Zhuhai 519000, China

**Keywords:** diabetes mellitus, dementia, cognitive function, mild cognitive impairment, Alzheimer disease, PPAR-γ agonists, meta-analysis

## Abstract

Diabetes mellitus (DM) is known to be a risk factor for dementia, especially in the elderly population, and close associations between diabetes and Alzheimer disease (AD) have been determined. Peroxisome proliferator-activated receptor-gamma (PPAR-γ) agonists are insulin-sensitising drugs. In addition to their anti-diabetic properties, their effectiveness in preventing and decreasing cognitive impairment are the most recent characteristics that have been studied. For this study, we conducted a systematic review and meta-analysis to critically analyse and evaluate the existing data on the effects of PPAR-γ agonist therapy on the cognitive status of patients. For this purpose, we first analysed both early intervention and later treatment with PPAR-γ agonists, according to the disease status. The involved studies indicated that early PPAR-γ agonist intervention is beneficial for patients and that high-dose PPAR-γ therapy may have a better clinical effect, especially in reversing the effects of cognitive impairment. Furthermore, the efficacy of pioglitazone (PIO) seems to be promising, particularly for patients with comorbid diabetes. PIO presented a better clinical curative effect and safety, compared with rosiglitazone (RSG). Thus, PPAR-γ agonists play an important role in the inflammatory response of AD or DM patients, and clinical therapeutics should focus more on relevant metabolic indices.

## 1. Introduction

Diabetes mellitus, which has a troublingly high prevalence, affects 463 million individuals throughout the world (International Diabetes Federation, 2019), and over one-quarter of the population aged ≥ 65 years. Diabetes mellitus (DM) is known to be a risk factor for dementia [1,2,3], especially in the elderly population. It has been reported to lead to inferior cognitive performance, when compared to age-matched healthy controls (HCs) [4]. Several meta-analyses have indicated that individuals with diabetes have a 73% increased risk of developing dementia and a 56% risk of developing Alzheimer disease [2], and research has suggested that type 2 diabetes mellitus (T2DM) results in a more rapid rate of cognitive decline than that typically associated with natural aging [5,6,7]. Furthermore, patients with pre-diabetes also have a higher risk for all-cause dementia and Alzheimer disease (AD) [3]. Diabetes is associated with AD through common pathophysiological mechanisms, such as increased levels of inflammatory indices, amyloid β-protein (Aβ) deposition, and increased oxidative stress [8]. Hence, aggressive management is necessary to prevent AD progression. Several studies have reported that anti-diabetic agents could potentially treat cognitive symptoms and modulate disease progression and cognitive decline in mild cognitive impairment (MCI)/AD [9]. Peroxisome proliferator-activated receptor-gamma (PPAR-γ) agonists are insulin-sensitising drugs designed for diabetes patients with insulin resistance; furthermore, they can regulate several cellular processes, such as Aβ degradation and the anti-inflammatory response [10,11]. In recent years, treatment of animal models of AD or MCI with thiazolidinediones, a PPAR-γ agonists, demonstrated an improvement in memory performance and a reduction in amyloid burden and inflammation [12,13,14,15]. Several observational studies and randomized controlled trials (RCTs) have also reported the effectiveness of PPAR-γ agonists in preventing and decreasing cognitive impairment. However, the reported results still present some considerable variabilities, and the effects of existing PPAR-γ agonists, in terms of preventing cognitive impairment, have not yet been summarized and analysed in observational research.

Hence, the primary objective of this study was to conduct a systematic review and meta-analysis to critically analyse and evaluate data on the effects of PPAR-γ agonist therapy on cognitive status. To this end, we first analysed both early intervention and later treatment with PPAR-γ agonists, according to the disease status. In addition, we comprehensively evaluated the efficacy and safety of PPAR-γ agonists in the treatment of MCI, mild to moderate AD, and AD based on clinical trials. 

## 2. Materials and Methods

### 2.1. Search Strategy

**Figure 1:**
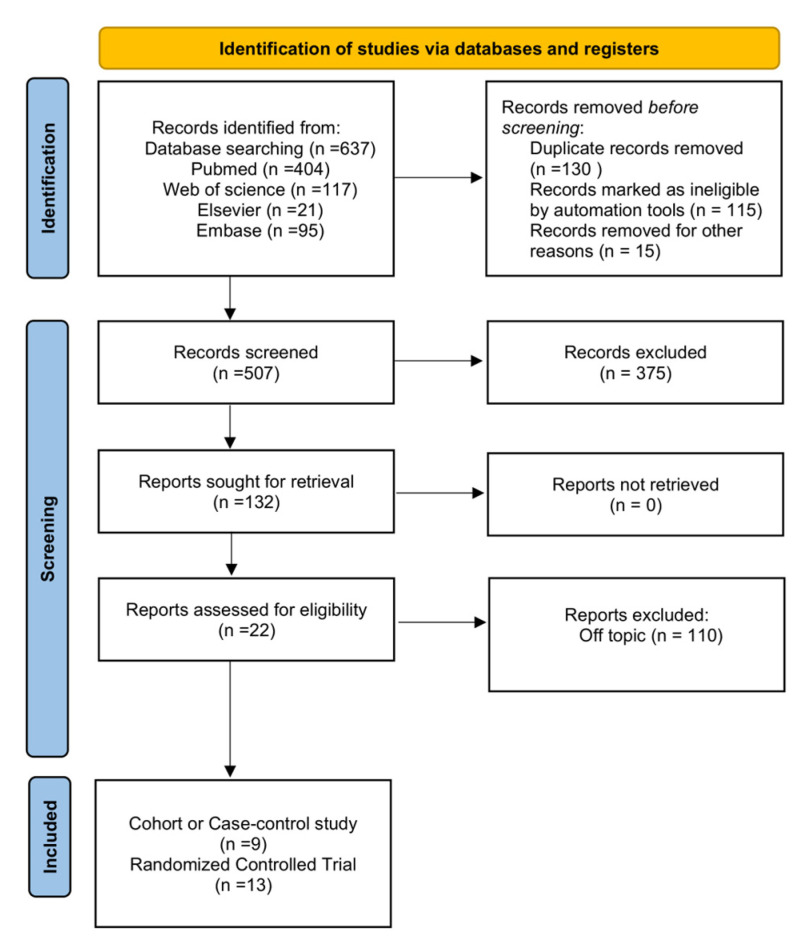
Flowchart of the meta-analysis study selection process.

We performed a literature search in July 2022 on the Elsevier, EMBASE, Web of Science, and PubMed databases. The following search terms were used: (1) “Cognitive impairment” or “Dementia” or “Alzheimer Disease”; (2) “PPAR-γ agonists/Peroxisome proliferator-activated receptor gamma agonists” or “Thiazolidinedione” or “Rosiglitazone or “pioglitazone”; (3) “the cohort studies” or “the Case-Control studies” or “the Randomized Controlled Trial studies”. In addition, the references of the retrieved papers and recent reviews were reviewed. The flow diagram of the search strategy is presented in Figure 1.

### 2.2. Study Criteria 

The inclusion criteria for studies were as follows: (1) cohort studies or case–control studies providing data on PPAR-γ (peroxisome proliferator-activated receptor-gamma) agonist treatments (“Treatment dose,” “Treatment duration,” “Effect on cognition, such as HR (hazard ratio),”); (2) validated diagnosis of “Cognitive impairment” or “Dementia” or “Alzheimer Disease”; (3) studies that provided information about PPAR-γ agonist treatments in cognitive impairment patients; and (4) articles that reported a clear comparison of PPAR-γ agonist treatments versus no PPAR-γ agonist treatments in population controls with a direct effect on cognitive or metabolic responses.

The exclusion criteria were as follows: (1) duplicate studies; (2) studies such as systemic reviews, meta-analyses, and comments; and (3) studies of PPAR-γ agonist treatments without detailed research data concerning clinical responses.

### 2.3. Data Extraction

The data extracted from each study included the first author’s name, the publication year, the country of study origin, the number of patients, median age, the neuropsychological response: Alzheimer Disease Assessment Scale Cognitive Score (ADAS-COG), Clinical Dementia Rating sum of boxes (CDR-SB), Digital symbol test (DST), Mini-Mental State Examination (MMSE), Rey Auditory Verbal Learning Test (RAVLT), metabolic response:fasting plasma glucose (FPG), homeostasis model assessment (HOMA %), Insulin, tumour necrosis factor (TNF-α), interleukin-6 (IL-6), C-reactive protein (CRP), adverse events (AEs), and HR. If a study did not clearly mention any of the above key points, it had not performed the required methods. Two of the authors (Hongfei Zhong and Qiuyun Tu) independently reviewed the selected studies and extracted the data. Discrepancies were resolved by discussion.

### 2.4. Statistical Analysis 

The data were abstracted and analysed using Stata (version 12), in order to make the outcomes more comprehensive. The binary variable outcomes were the AEs (anaemia, peripheral oedema, bone fractures, cardiac failure, diarrhoea, dizziness, hepatic disorders, headache, hyperlipidaemia, hypoglycaemia, insomnia, muscle pain, nasopharyngitis, nausea), and the data are expressed as a RR (risk ratio) with 95% CI (confidence interval). The effect was estimated using a random effects model. Other continuous variable outcomes were the rating scale (ADAS-COG, CDR-SB, DST, MMSE, and RAVLT), and the metabolic response (FPG, HOMA%, Insulin, TNF-α, IL-6, CRP). Data are expressed as the SMD (standardized mean difference) with 95% CI. In addition, we performed a random-/fixed-effect meta-analysis using maximally adjusted HRs with 95% CI for the observational studies. When combining studies, the random effects model was used to account for study heterogeneity. We used the Q statistic and *I^2^* tests to evaluate the heterogeneity. Low, moderate and high heterogeneities were represented by thresholds of <25%, 25–75%, and >75%, respectively. *p* ≤ 0.05 was considered significant in all statistical tests.

### 2.5. Data Analysis 

The binary variable outcomes were the incidence of the AEs (anaemia, peripheral oedema, bone fractures, cardiac failure, diarrhoea, dizziness, hepatic disorders, headache, hyperlipidaemia, hypoglycaemia, insomnia, muscle pain, nasopharyngitis, nausea). In addition, the data of dementia risk are expressed as the hazard ratio (HR) or odds ratio (OR) with 95% confidence interval (CI); the estimation of the effect was performed using a random effects model. 

## 3. Results

A total of 22 studies were included in this article, with 13 controlled trials [16,17,18,19,20,21,22,23,24,25,26,27,28] and nine observational studies [29,30,31,32,33,34,35,36,37] included in the quality systematic review after 637 registered studies were assessed. Nine articles with a total of 433,823 DM (diabetes mellitus) patients were included in the observational studies (Table 1), and the association between PPAR-γ agonist intake and dementia risk was examined. In the RCT studies, the outcomes of the neuropsychological response, metabolic response, and AEs after treatment with PPAR-γ drugs in cognitive impairment patients were reported (Table 2).

### 3.1. Effect of PPAR-γ Agonists on Cognition Based on the Observational Studies

**Figure 2:**
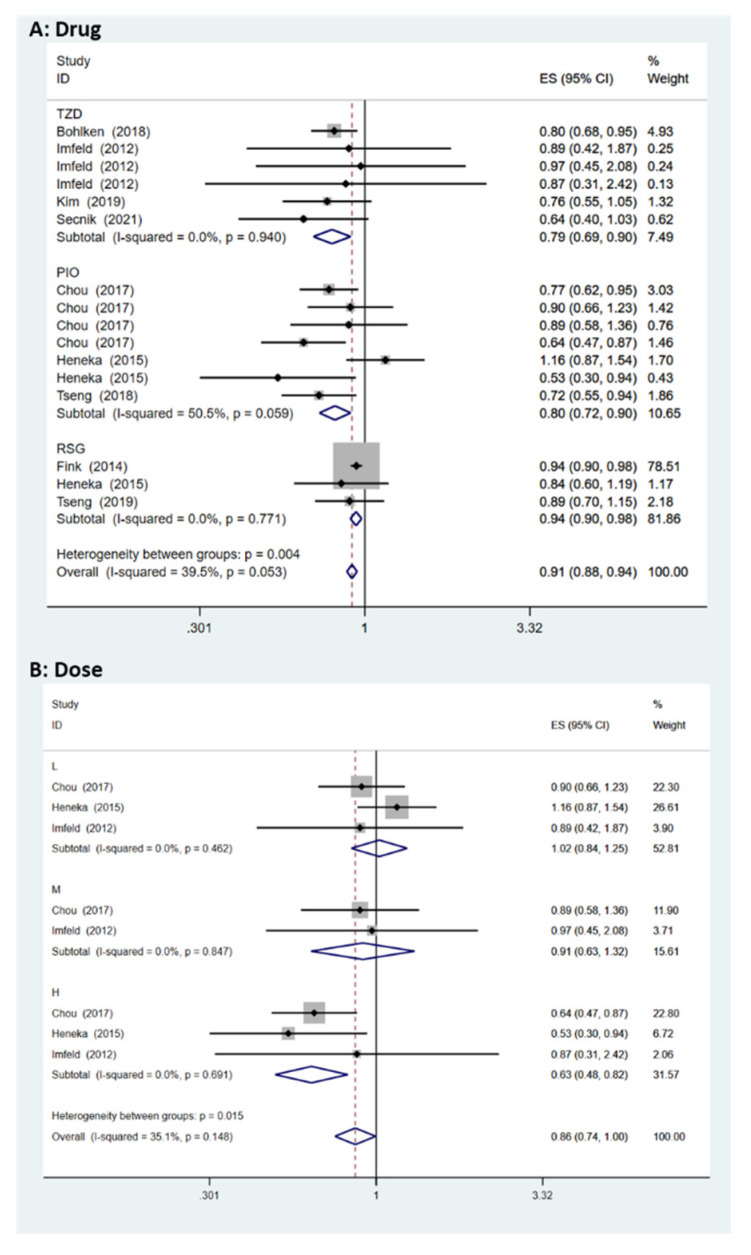
Dementia risk under PPAR-γ agonist treatment in DM patients (sub-group: Figure 2A drug, Figure 2B drug dose) [29,30,31,32,33,34,35,36,37].

**Figure 3:**
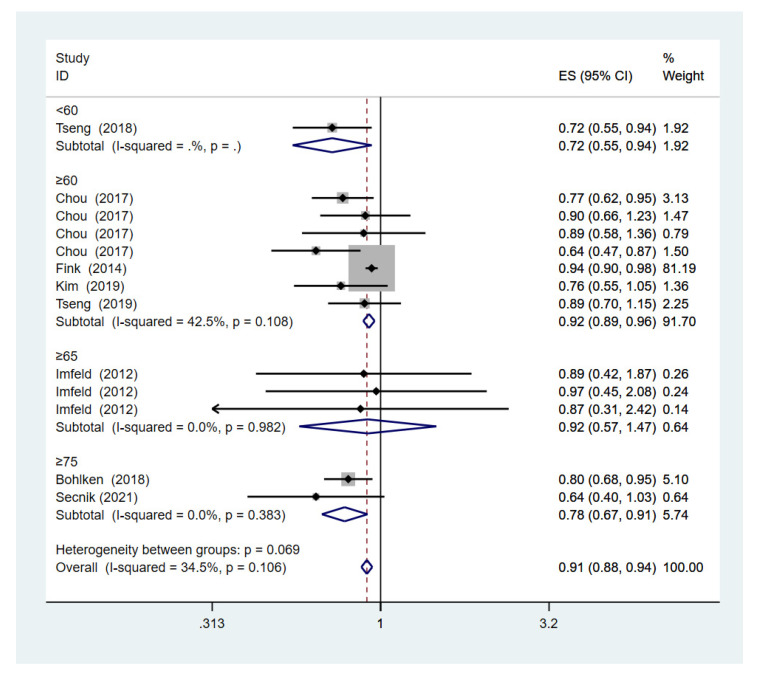
Dementia risk under PPAR-γ agonist treatment in DM patients (sub-group: age) [29,30,31,33,34,35,36].

Nine observational studies were included (two case–control studies and seven cohort studies) in the meta-analysis, where the HR for observational studies was equal to 0.91 (95% CI = [0.88, 0.94], *I^2^* = 39.5%) in the random-effect analysis (Figure 2A). In the sub-group analysis of drugs, a significant HR = 0.79 (95% CI = [0.69, 0.90], *I^2^* = 0%) was found for TZD (thiazolidinedione). The figures for PIO and RSG in the sub-group analysis were HR = 0.80 (95% CI = [0.72, 0.90], *I^2^* = 50.5%) and HR = 0.94 (95% CI = [0.90, 0.98], *I^2^* = 0%), respectively (Figure 2A). In additional, a dose–response relationship was observed in the sub-group of drug dose (Figure 2B), L: HR = 1.02 (95% CI = [0.84, 1.25], *I^2^* = 0%), M: HR = 0.91 (95% CI = [0.63, 1.32], *I^2^* = 0%), and H: HR = 0.63 (95% CI = [0.48, 0.82], *I^2^* = 0%). Moreover, the age sub-group results are shown in Figure 3. The region sub-group analysis included Europe (HR = 0.88, 95% CI = [0.78, 0.99], *I^2^* = 34.4%) and Asia (HR = 0.79, 95% CI = [0.71, 0.87], *I^2^* = 0%).

### 3.2. Effect of PPAR-γ Agonists on Cognition Based on Clinical Trials

In 13 double-blind randomized controlled studies, the effect of PPAR-γ agonists versus placebo on cognitive performance was evaluated in 5102 cognitive impairment patients with and without DM.

#### 3.2.1. Neuropsychological Outcomes/Neuropsychological Scales

Neuropsychological outcomes were measured in all studies, mainly including ADAS-Cog, CDR-SB, DST, MMSE, and RAVLT. Details of the outcomes are listed in Table 3; due to the complexity of the source of the scales, the data are expressed as the SMD (standardized mean difference) with 95% CI (confidence interval). Furthermore, the sub-group analysis considered drug, dose, disease status, treatment duration, and region. 

#### 3.2.2. Metabolic Outcomes

We investigated the glycometabolism, lipid metabolism, and inflammatory response in MCI or AD patients after PPAR-γ agonist treatments, data are expressed as the SMD (standardized mean difference) with 95% CI, and the metabolic outcomes in studies have the same unit (FPG: mg/dL; HOMA%: -; insulin: μU/mL; TNF-α: pg/mL; IL-6: pg/mL; CRP: mg/L), as detailed in Table 3. FPG was decreased after PPAR-γ agonist treatments, but the HOMA % and insulin were not significantly changed. As for the inflammatory response, three related indices were considered in our study, and we found that TNF-α was significantly decreased, while IL-6 and CRP were not changed.

#### 3.2.3. AEs

We investigated all types of AEs. A total of 14 subjects were included in the study (anaemia, peripheral oedema, bone fractures, cardiac failure, diarrhoea, dizziness, hepatic disorders, headache, hyperlipidaemia, hypoglycaemia, insomnia, muscle pain, nasopharyngitis, and nausea), as shown in Table 4. The sub-group analysis of any AEs included drug, dose, disease status, and treatment duration. Among the 14 AE categories, below are some of the high-risk AEs: anaemia (RR = 5.96, 95% CI = [3.25,10.95], *I^2^* = 35.3%), peripheral oedema (RR = 4.35, 95% CI = [3.29,5.75], *I^2^* = 79.9%), and hyperlipidaemia (RR = 4.98, 95% CI = [1.71,14.50], *I^2^* = 6.8%).

## 4. Discussion

The published research on PPAR-γ agonist therapy has mainly focused only on RCT clinical studies. Liu and Cheng et al. have taken PPAR-γ agonists as sufficient evidence to treat cognitive impairment patients [38,39]. However, various data were not comprehensively assessed, such as treatment dosing, duration, neuropsychological scales, and metabolism. In recent years, more studies have reported updated research on PPAR-γ agonist treatments. In 2021, Burns et al. [16] conducted a phase 3, randomized, double-blind, placebo-controlled trial which enrolled 3494 participants. Cohort and case–control studies have also been published in recent years. Khan et al. [40] and Jojo et al. [41] have updated the current progress on PPAR-γ agonists as an emerging therapeutic approach for the treatment of Alzheimer’s disease. Hence, updating related meta-analysis research is necessary. 

In this systematic review, we found that the available evidence provides some support for PPAR-γ agonists having a protective effect against dementia in individuals who are taking them for the management of diabetes. Furthermore, the considered studies have deeply discussed the clinical efficacy of PPAR-γ agonists versus placebo in clinical trials, including the dose–response effect, treatment duration, neuropsychological scales, metabolic outcomes, and full AE analysis.

Observational studies have shown that PPAR-γ agonists have a protective effect against cognitive decline in diabetic patients (HR = 0.91, 95% CI = [0.88, 0.94] and *I^2^* = 39.5%), where the heterogeneity of the data was acceptable. These results suggest that early PPAR-γ agonist drug intervention in DM patients has potential benefits. Compared with RSG, PIO presented a better prophylactic effect (Figure 2A). At present, there are two major glitazones: rosiglitazone and pioglitazone. Pioglitazone significantly reduces the neuroinflammation response and cerebral oxidative stress and, so, might play a neuroprotective role associated with improvements in inflammation-related neuropathy and insulin signalling pathways. Additionally, Yang et al. demonstrated that treatment with pioglitazone (20 mg/kg; intragastric administration) ameliorated Aβ42 deposition in the insulin-resistant (IR) rat hippocampus by increasing insulin-degrading enzymes (IDE) and PPAR-γ expression, as well as ameliorating Aβ accumulation via the AKT/GSK3β signalling pathway [42], and Aβ accumulation was measured by hippocampus immunohistochemistry. Pioglitazone not only recovered the memory and cognitive deficits, but also ameliorated Aβ deposition. The involved studies indicated that high-dose PPAR-γ agonists may play protective roles in DM patients. Furthermore, DM patients less than 60 have a lower risk of dementia (HR = 0.72, 95% CI = [0.55, 0.94]), suggesting that early PPAR-γ agonist intervention in DM patients has potential benefits. According to the geographical distribution sub-group, Asian DM patients may have a lower risk of dementia with early PPAR-γ agonist intervention. 

**Figure 4:**
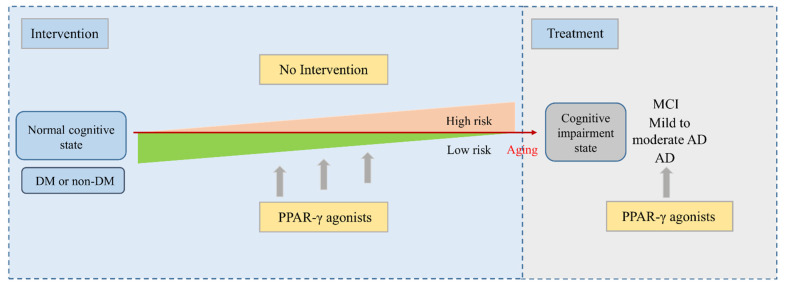
PPAR-γ agonist treatment plays an important role in disease progression (early intervention and later treatment).

The established evidence indicated a considerable overlap in the putative pathophysiological mechanisms for DM and cognitive impairment and dementia [43],such as increased levels of inflammatory indices, amyloid β-protein (Aβ) deposition, and increased oxidative stress. The epidemiological trends for dementia are very similar to those observed in DM [44]. Epidemiological studies have established an increased risk of dementia among individuals with diabetes mellitus [45], and type 2 diabetes mellitus (T2DM) might also be a potential risk for MCI progressing into AD, through the induction of oxidative and inflammatory stress and vascular dysfunction [46]. Studies have also shown a positive association between DM and mild cognitive impairment (MCI), and an accelerated progression from MCI to dementia in DM [47]. Hence, early intervention by using hypoglycaemic agents may be beneficial for reducing the risk of dementia, as shown in Figure 4.

Furthermore, evidence from various studies has suggested that insulin sensitizers (TZDs) are key therapeutic targets in MCI and AD patients, with an associated Aβ degradation and anti-inflammatory response [10,11]. Hence, we collected the RCTs in the above disease which were treated with PPAR-γ agonists (Figure 4), and analysed the clinical effects from the perspective of drug treatment. We enrolled all outcomes, including neuropsychological outcomes, metabolic outcomes, and AEs.

In the neuropsychological outcomes of the clinical trial studies, we measured the cognitive indices, including ADAS-Cog, CDR-SB, DST, MMSE, and RAVLT. Compared with previous studies [38,39], we used the SMD (standardized mean difference) to reduce the complexity regarding the sources of the scales. Eight studies measured the changes in ADAS-Cog (Table 2). In the meta-analysis, a significant change in ADAS-Cog score was observed (SMD = −0.07, 95% CI [−0.13, −0.01], *I^2^* = 11.5%, *p* = 0.322). In the sub-group analyses, four studies compared PIO with placebo, and there was a significant change in ADAS-Cog score (SMD = −0.39, 95% CI [−0.70, −0.08], *I^2^* = 28.6%, *p* = 0.220), but the RSG was ineffective, compared with PIO (SMD = −0.06, 95% CI [−0.12, 0.00], *I^2^* = 0%, *p* = 0.777), although there was no heterogeneity. Therefore, PIO may be beneficial to delay AD progression. Furthermore, AD patients with DM seemed to have the best treatment benefits (SMD = −0.82, 95% CI [−1.35, −0.28], *I^2^* = 33.3%, *p* = 0.221); see Table 3. In the dose sub-group, PPAR-γ agonist therapy may have dose-dependent effects, where increasing the dosage of the PPAR-γ agonist may lead to lower ADAS-Cog clinical scores (L/Low dose/2 mg: SMD = −0.08, 95% CI [−0.16, 0.00], *I^2^* = 0%; H/High dose/15 mg: SMD = −0.39, 95% CI [−0.70, −0.08], *I^2^* = 28.6%), meaning AD patients using a higher dose of PPAR-γ agonist therapy may have a better therapeutic outcome. We also considered a dose sub-group in the treatment duration, which indicated that PPAR-γ agonists may have a dose-dependent effect in 24-week therapy (Table 3). CDR-SB presented no significant difference after treatment with PIO or RSG (overall: SMD = −0.06, 95% CI [−0.14, 0.01], *I^2^* = 43.3%; PIO: SMD = 0.22, 95% CI [−0.14, 0.58], *I^2^* = 0%; RSG: SMD = −0.07, 95% CI [−0.15, 0.00], *I^2^* = 60.8%). According to the region sub-group analysis, PPAR-γ agonists may lead to lower ADAS-Cog clinical scores in Asian patients (SMD = −0.73, 95% CI = [−1.16, −0.30]), while CDR-SB scores showed no significant differences. In early MCI patients, the DST score increased (SMD = 1.01, 95% CI = [0.78, 1.42]), as presented by Hildreth and Ryan et al. [22,24]; DST reflects early arithmetic ability and, so, in early cognitive impairment patients, early intervention with PPAR-γ agonists may effectively better reverse the loss in arithmetic cognitive ability. Unfortunately, due to the small number of studies reporting MMSE and RAVLT scores, we found that there was no apparent effect of PPAR-γ agonists on MMSE (SMD = 0.37, 95% CI = [−0.09, 0.83], *I^2^* = 0%) or RAVLT (SMD = −0.15, 95% CI = [−0.44, 0.13], *I^2^* = 0%) scores.

Six metabolic outcomes were investigated in our study. FPG (SMD = −0.07, 95% CI = [−0.13, −0.01], *I^2^* = 0%) and TNF-α (SMD = −0.60, 95% CI = [−1.04, −0.15], *I^2^* = 19.8%) were significantly changed after PPAR-γ agonist treatment. The most significant metabolic change was the inflammatory response. In DM and AD mouse models, chronic treatment of pioglitazone (15 and 30 mg/kg) significantly attenuated TNF-α and IL-6, compared to an Aβ-treated group (*p* < 0.05), the increase in IL-6 and TNF-α expression which indicates the involvement of inflammatory activity, pioglitazone significantly reduced the neuroinflammation response (e.g., reducing the inflammatory cytokines TNF-a, IL-6, IL-8, and so on) and cerebral oxidative stress and, thus, might play a neuroprotective role associated with improvements in inflammation-related neuropathy and insulin signalling pathways [48,49]. Studies have also reported that rosiglitazone treatment may decrease the inflammatory markers TNF- α and IL-6a [15,50,51]. Furthermore, as investigated in the meta-analysis, TNF-α decreased after PPAR-γ agonist treatment (SMD= −0.60, 95%CI = [−1.04, −0.15], *I^2^* = 19.8%). The inflammatory response may be crucial in the AD progression of DM patients. The involved studies indicated that the anti-inflammatory properties of PPAR-γ agonists may have potential roles in the treatment of AD. 

We also discussed the AEs during PPAR-γ agonist treatment, and found that the risk of suffering from AEs increased with the dose and duration of medication (Table 4). Other AEs, such as anaemia and peripheral oedema, presented the same tendency. Previous articles have reported that PIO treatment may lead to a higher risk of AEs; in their sub-group analyses, a borderline result was detected in pioglitazone- versus placebo-treated subjects (RR =7.43, 95 % CI = [0.96, 57.5]) [38]. This high risk of AEs may induce clinical designers to conduct lesser clinical trials associated with PIO. In contrast, the present study indicated that patients treated with PIO seem to have a lower risk of AEs (RR = 0.21, 95% CI = [0.17, 0.25], *I^2^* = 85.0%), compared with RSG (RR = 1.06, 95% CI = [1.01, 1.11], *I^2^* = 57.3%). Only five studies were included in the previous analysis, with the number of included patients being 2127. A phase 3, randomised, double-blind, placebo-controlled trial (TOMMORROW) enrolled 3494 participants, with 3465 participants included in the safety analysis. In the present meta-analysis, we enrolled these published studies, and PIO seemed to have a lower risk of AEs, compared with RSG. Anaemia, peripheral oedema and hyperlipidaemia were the highest risk AEs (anaemia RR = 5.96, 95% CI = [3.25, 10.95], *I^2^* = 35.3%; peripheral oedema RR = 4.35, 95% CI = [3.29, 5.75], *I^2^* = 79.9%; hyperlipidaemia RR = 4.98, 95% CI = [1.71, 14.50], *I^2^* = 6.8%). Clinicians must pay attention to these safety events. 

We must note that there were some limitations of our study. First, several clinical studies on PPAR-γ agonist therapy are still ongoing [52,53,54], and we could not enrol these yet-unpublished studies. Second, some of the studies that were included failed to directly present concrete data in the text, such as changes in standard deviation and mean values from baseline; in order to ensure the reliability of the data, we bypassed the second transformation entirely, which may have led to a reduced amount of data. Third, as for the AE data, a large number of data were included; however, for the neuropsychological outcomes, the data are still limited. We expect that more RCTs will help us to expand the sample size and make a more accurate conclusion in the future. Fourth, in the observational studies, the time span of the drug intervention was huge and the data were limited, from 45–90 days to 11 years and, thus, a sub-group analysis of the intervention time could not be performed. Due to this, further studies using larger samples and detailed intervening measures are required to provide clearer results.

## 5. Conclusions

This systematic review and meta-analysis study indicated the beneficial effects of PPAR-γ agonists on the improvement of cognitive function in cross-sectional and cohort studies, as well as RCTs. We divided these results into two parts: PPAR-γ agonist intervention and therapy. Cognitive impairment is a progressive disease, with patients suffering progressive deterioration of cognitive and functional skills and having difficulties with memory, language, problem-solving, and other thinking skills. Such diseases include MCI, mild to moderate AD, and AD. Continuous monitoring is essential during PPAR-γ agonist treatment. The enrolled studies indicated that early PPAR-γ agonist intervention is beneficial for patients, and that high-dose PPAR-γ therapy may have a better clinical effect, especially in the reversing the effects of cognitive impairment. Furthermore, the efficacy of PIO seems to be promising, particularly for patients with comorbid diabetes. PIO presented a better clinical curative effect and safety, compared with RSG, and it deserves more well-designed trials with large sample sizes in the future. PPAR-γ agonists play an important role in the inflammatory response of AD or DM patients, and clinical therapeutics should focus more on relevant metabolic indices.

## Figures and Tables

**Table 1:** Characteristics of included observational studies.

Design/Reference	Participants			Drugs			Outcome Measure	Effect on Cognition
	Number	Median Age	Basic Disease	Name	Treatment Dose	Duration		
Case–Control [29]	8276	79.7	DM	Glitazone	NR	NR	(ICD-10: F01, F03, G30)	OR = 0.80 [0.68–0.95]
Cohort [30]	19,203	≥60	DM	pioglitazone	>0.83 DDDs	>252 days	ICD-9-CM	pioglitazone user HR = 0.77 [0.62–0.95] <0.83 DDDs HR = 0.90 [0.66–1.23] 0.83–1.00 DDDs HR = 0.89 (0.58–1.36) >1.00 DDDs HR = 0.64 (0.47–0.87)
Cohort [31]	145,717	60	DM	Rosiglitazone	NR	NR	NR	HR = 0.94, *p* = 0.004
Cohort [32]	145,928	≥60	DM and non-DM	Rosiglitazonepioglitazone	NR	NR	Tenth Revision [ICD-10]	Diabetes & PIO < 8 HR = 1.16 [0.87–1.55] Diabetes & PIO ≥ 8 HR = 0.53 [0.30–0.94] Rosiglitazone HR = 0.84 [0.59–1.18]
Case–Control [33]	7086	≥65	DM	Thiazolidinedione	Prescriptions 1–9 10–9 ≥30	45–90 days	NR	Thiazolidinedione prescriptions 1–9 OR = 0.89 [0.42–1.86] 10–9 OR = 0.97 [0.45–2.07] ≥30 OR = 0.87 [0.31–2.40]
Cohort [34]	91,219	≥60	T2DM	Thiazolidinedione	NR	11 years	Tenth Revision [ICD-10]	Dementia HR=0.79 [0.77, 0.81] Alzheimer’s dementia HR = 0.80 [0.77, 0.83] Vascular dementia HR = 0.78 [0.75, 0.82] Thiazolidinedione HR = 0.76 [0.55, 1.05]
Cohort [35]	335	79.7	DM	Thiazolidinedione	NR	NR	Tenth Revision [ICD-10]	HR = 0.64 [0.40–1.03]
Cohort [36]	11,011	58.7	T2DM	pioglitazone	NR	20 months	Tenth Revision [ICD-10]	HR = 0.72 [0.54–0.94]
Cohort [37]	5048	61.2	T2DM	Rosiglitazone	NR	NR	Tenth Revision [ICD-10]	HR = 0.89 [0.69–1.15]

Abbreviation: DM, diabetes mellitus; T2DM, type 2 diabetes mellitus; ICD, international classification of diseases; DDDs, defined daily doses; HR, hazard ratio; OR, odds ratio; NR, not reported.

**Table 2:** Characteristics of included controlled trials.

Design/Reference	Participants				Intervention		Drugs		Outcome Measure		AEs
	Number	Median Age	Cognitive Function	APOE- 4	Control	Case	Dose	Duration	Cognitive	Metabolic	
RCT [16]	56	74.5	MCI	NR	1507	1531	PIO 0.8 mg/day	6 months	MMSE	NR	√
RCT [17]	29	74.9	AD	NR	15	14	PIO 15 mg	6 months 12 months 18 months	ADAS-Cog NPI NOSGER ADFACS CDR-SB	NR	√
RCT [18]	693	72.5	Mild to moderate AD	√	165	166	PIO 2/8/10 mg	24 weeks	MMSE ADAS-Cog	NR	√
RCT [19]	32	76.5	12 AD/3 MCI	NR	17	15	PIO 15–30 mg	6 months	MMSE ADAS-Cog WMSR	FPG Insulin HOMA%	√
RCT [20]	34	77	mild AD with T2DM	√	17	17	PIO 15–30 mg	6 months	ADAS-Cog	TNF CRP IL-6	NR
RCT [21]	1393	74.1	AD	√	496	494	RSG 2, 8 mg	48 weeks	MMSE ADAS-Cog CDR-SB	NR	√
RCT [21]	1429	73.2	AD	√	487	790	RSG 2, 8 mg	48 weeks	MMSE ADAS-Cog CDR-SB	NR	√
RCT [22]	49	66.0	MCI	√	25	25	RSG 30 mg	6 months	MMSE ADAS-Cog DST	TNF FPG Insulin CRP IL-6	NR
RCT [23]	511	70.7	Mild to moderate AD	NR	122	131	RSG 2, 4, 8 mg	24 weeks	ADAS-Cog MMSE	NR	√
RCT [24]	145	60.2	T2DM	NR	70	66	RSG 4 mg	4 weeks	DST SWMR RAVLT	Insulin FPG HOMA %	NR
RCT [25]	42	77.5	mild AD DM	NR	21	21	PIO 15–30mg	6 months	MMSE ADAS-cog WMSR	FPG Insulin HOMA%	NR
RCT [26]	80	72.2	Mild to moderate AD	NR	29	31	RSG 8 mg	12 months	ADAS-cog	FDG-PET	NR
RCT [27]	30	73.0	AD	NR	10	20	RSG 4 mg	6 months	MMSE	Insulin FPG	NR
RCT [28]	579	NR	Mild to moderate AD	√	165	166	RSG 2, 8 mg	24 weeks	NR	NR	√

Abbreviation: RCT, randomized double-blind controlled clinical trial (RCT); MCI, mild cognitive impairment; AD, Alzheimer’s disease; PIO, pioglitazone; RSG, rosiglitazone; DM, diabetes mellitus; T2DM, type 2 diabetes mellitus; MMSE, Mini-Mental State Examination; ADAS-COG, Alzheimer Disease Assessment Scale Cognitive Score; CDR-SB, Clinical Dementia Rating sum of boxes; NOSGER, Nurse’s Observation Scale for Geriatric Patients; NPI, Neuropsychiatric Inventory; WMSR, Wechsler Memory Scale Revised; DST, Digital symbol test; RAVLT, Rey Auditory Verbal Learning Test; CIBIC+, Clinician’s Interview-Based Impression of Change plus Carer Interview; FDG-PET, Positron emission tomography (PET) with 2-fluoro-2-deoxy-D-glucose; HOMA%, homeostasis model assessment; FPG, fasting plasma glucose; TNF, tumour necrosis factor; CRP, C-reactive protein; IL-6, interleukin-6; NR, not reported.

**Table 3:** Neuropsychological and metabolic outcomes.

Neuropsychological	Results			
		SMD	95% CI	*I^2^*
1. ADAS-COG		−0.07	−0.13, −0.01	11.5%
Drug	PIO	−0.39	−0.70, −0.08	28.6%
	RSG	−0.06	−0.12, 0.00	0%
Dose	L	−0. 08	−0.16, 0.00	0%
	M	−0.03	−0.12, 0.06	0%
	H	−0.39	−0.70, −0.08	28.6%
Disease	Mild to moderate AD	−0.05	−0.12, 0.01	0%
	AD with DM	−0.82	−1.35, −0.28	33.3%
	AD	−0.11	−0.27, 0.05	0%
Duration	24 weeks	−0.11	−0.21, 0.00	31.8%
	L	−0.05	−0.22, 0.12	0%
	M	−0.09	−0.26, 0.09	0%
	H	−0.53	−0.90, −0.16	43.2%
	48 weeks	−0.05	−0.12, 0.02	0%
	L	−0.09	−0.19, 0.01	46.7%
	M	−0.01	−0.11, 0.09	0%
	H	−0.13	−0.91, 0.64	-
	72 weeks	−0.04	−0.82, 0.75	-
	M	0.29	−0.22, 0.80	-
	H	−0.04	−0.82, 0.75	-
Region	America	−0.06	−0.12, −0.00	0%
	Europe	0.29	−0.22, 0.80	-
	Asia	−0.73	−1.16, −0.30	0%
2.CDR-SB		−0.06	−0.14, 0.01	43.3%
Drug	PIO	0.22	−0.14, 0.58	0%
	RSG	−0.07	−0.15, −0.00	60.8%
Dose	L	−0.14	−0.25, −0.04	72.0%
	M	−0.00	−0.11, 0.10	0%
	H	0.22	−0.14, 0.58	0%
Disease	Mild to moderate AD	−0.07	−0.15, −0.00	60.8%
	AD with DM	0.40	−0.21, 1.02	-
	AD	0.12	−0.33, 0.57	0%
Duration	24 weeks	0.17	−0.30, 0.65	28.6%
	48 weeks	−0.07	−0.14, −0.00	59.6%
	72 weeks	0.04	−0.74, 0.83	-
Region	America	−0.07	−0.14, 0.00	40.4%
	Europe	0.40	−0.21, 1.02	-
3. DST		1.01	0.78, 1.42	95.7%
4. MMSE		0.37	−0.09, 0.83	0%
5. RAVLT		−0.15	−0.44, 0.13	0%
**Metabolic**	**Results**			
	**SMD**		**95% CI**	***I^2^***
1. FPG (mg/dL)	−0.07		−0.13, −0.01	0%
2. HOMA %(-)	0.13		−0.14, 0.41	85.5%
3. Insulin (μU/mL)	0.02		−0.29, 0.32	80.4%
4. TNF-α (pg/mL)	−0.60		−1.04, −0.15	19.8%
5. IL-6 (pg/mL)	−0.30		−0.73, 0.14	0%
6. CRP (mg/L)	−0.18		−0.62, 0.25	73.4%

Abbreviation: MCI, mild cognitive impairment; AD, Alzheimer’s disease; PIO, pioglitazone; RSG, rosiglitazone; DM, diabetes mellitus; T2DM, type 2 diabetes mellitus; MMSE, Mini-Mental State Examination; ADAS-COG, Alzheimer Disease Assessment Scale Cognitive Score; CDR-SB, Clinical Dementia Rating sum of boxes; DST, Digital symbol test; RAVLT, Rey Auditory Verbal Learning Test; HOMA %, homeostasis model assessment; FPG, fasting plasma glucose; TNF, tumour necrosis factor; CRP, C-reactive protein; IL-6, interleukin-6; SMD, standard mean difference; CI, confidence interval.

**Table 4:** Adverse events during PPAR-γ agonist treatments.

AEs	Results				
		RR	95% CI	*I^2^*	Risk
1. Any AEs		0.82	0.78, 0.86	96.9%	
Drug	PIO	0.21	0.17, 0.25	85.0%	
	RSG	1.06	1.01, 1.11	57.3%	
Dose	0.8 mg	0.98	0.94, 1.03	-	
	2 mg	1.02	0.99, 1.05	0%	
	15 mg	7.42	0.96, 57.44	0%	
Duration	24 weeks	0.97	0.88, 1.06	57.7%	
	48 weeks	1.05	1.00, 1.11	74.8%	
	72 weeks	9.60	0.56, 163.58	-	
Disease	Mild to moderate AD	1.06	1.01, 1.12	28.3%	
	AD with DM	7.00	0.38, 127.69	-	
	AD	0.35	0.30, 0.40	98.2%	
2. Anaemia		5.96	3.25, 10.95	35.3%	high
Drug	PIO	1.61	0.31, 8.24	-	
	RSG	6.80	3.51, 13.18	28.8%	
3. Peripheral oedema		4.35	3.29, 5.75	79.9%	high
Dose	2 mg RSG	2.31	1.50, 3.54	72.7%	
	8 mg RSG	6.39	4.36, 9.38	72.0%	
4. Bone fractures		0.86	0.67, 1.11	0%	
5. Cardiac failure		0.66	0.34, 1.29	0%	
Drug	PIO	0.62	0.30, 1.28	-	
	RSG	1.00	0.14, 7.04	0%	
6. Diarrhoea		1.05	0.78, 1.42	0%	
7. Dizziness		1.19	0.84, 1.68	0%	
8. Hepatic disorders		0.61	0.38, 0.98	0%	
9. Headache		0.77	0.40, 1.48	38.3%	
10. Hyperlipidaemia		4.98	1.71, 14.50	6.8%	high
11. Hypoglycaemia		0.81	0.55, 1.19	0%	
12. Insomnia		0.64	0.23, 1.79	0%	
13. Muscle pain		3.26	1.81, 5.87	37.9%	
14. Nasopharyngitis		1.23	0.92, 1.65	0%	
15. Nausea		2.07	0.75, 5.70	0%	

Abbreviation: AEs, adverse events; PIO, pioglitazone; RSG, rosiglitazone; AD, Alzheimer’s disease; DM, diabetes mellitus; RR, Risk ratio; CI, confidence interval.

## Data Availability

The data sets used and analysed during the current study are available from the corresponding author.

## Abbreviations

RCT: randomized double-blind controlled clinical trial; MCI, mild cognitive impairment; AD, Alzheimer’s disease; PIO, pioglitazone; RSG, rosiglitazone; TZD, thiazolidinedione; DM, diabetes mellitus; T2DM, type 2 diabetes mellitus; MMSE, Mini-Mental State Examination; ADAS-COG, Alzheimer Disease Assessment Scale Cognitive Score; CDR-SB, Clinical Dementia Rating sum of boxes; NOSGER, Nurse’s Observation Scale for Geriatric Patients; NPI, Neuropsychiatric Inventory; WMSR, Wechsler Memory Scale Revised; DST, Digital symbol test; RAVLT, Rey Auditory Verbal Learning Test; CIBIC+, Clinician’s Interview Based Impression of Change plus Carer Interview; FDG-PET, Positron emission tomography (PET) with 2-fluoro-2-deoxy-D-glucose; HOMA %, homeostasis model assessment; FPG, fasting plasma glucose; TNF, tumour necrosis factor; CRP, C-reactive protein; IL-6, interleukin-6; NR, not report; PPAR-γ, peroxisome proliferator-activated receptor-gamma; HCs, healthy controls; AEs, adverse events; MD, mean difference; SMD, standard mean difference; CI, confidence interval; ICD, International Classification of Diseases; DDDs, defined daily doses; RR, risk ratio; HR, hazard Ratio; OR, odds ratio; NR, not reported.
